# High Glucose Enhances the Odonto/Osteogenic Differentiation of Stem Cells from Apical Papilla via NF-KappaB Signaling Pathway

**DOI:** 10.1155/2019/5068258

**Published:** 2019-04-08

**Authors:** Yanping Wang, Yanqiu Wang, Yadie Lu, Jinhua Yu

**Affiliations:** ^1^Endodontic Department, the Affiliated Stomatological Hospital of Soochow University, Suzhou Stomatological Hospital, 1505 Renmin Road, Suzhou, Jiangsu 215005, China; ^2^Jiangsu Key Laboratory of Oral Diseases, Nanjing Medical University, 136 Hanzhong Road, Nanjing, Jiangsu 210029, China; ^3^Endodontic Department, School of Stomatology, Nanjing Medical University, 136 Hanzhong Road, Nanjing, Jiangsu 210029, China; ^4^Endodontic Department of the West Branch of Hangzhou Dental Hospital, 306 Tianmushan Road, Hangzhou, Zhejiang 310013, China

## Abstract

**Objective:**

The transport and metabolism of glucose are important during mammalian development. High glucose can mediate the biological characteristics of mesenchymal stem cells (MSCs). However, the role of high glucose in the odonto/osteogenic differentiation of stem cells from apical papilla (SCAPs) is unclear.

**Materials and Methods:**

SCAPs were isolated and identified* in vitro*. Then, SCAPs were cultured in normal *α*-MEM and high glucose *α*-MEM separately. MTT assay was applied to observe the proliferation of SCAPs. ALP activity, alizarin red staining, real-time RT-PCR, and western blot were used to detect the odonto/osteogenic capacity of SCAPs as well as the participation of NF-*κ*B pathway.

**Results:**

SCAPs in 25mmol/L glucose group expressed the maximum proteins of RUNX2 and ALP as compared with those in 5, 10, and 15 mmol/L groups. MTT assay showed that 25 mmol/L glucose suppressed the proliferation of SCAPs. ALP assay, alizarin red staining, real-time RT-PCR, and western blot showed 25 mmol/L high glucose can obviously enhance the odonto/osteogenic capacity of SCAPs. Moreover, the NF-*κ*B pathway was activated in 25mmol/L glucose-treated SCAPs and the odonto/osteogenic differentiation was inhibited following the inhibition of NF-*κ*B signaling pathway.

**Conclusions:**

High glucose can enhance the odonto/osteogenic capacity of SCAPs via NF-*κ*B pathway.

## 1. Introduction

Stem cells from apical papilla (SCAPs), derived from the developing apical complexes, are considered as the attractive candidates for tooth and bone regeneration [1, 2]. They contain the ability to modulate the dental root development and play a key role in the regenerative endodontic procedures in immature teeth with pulp necrosis [3]. Previous studies have revealed that the odonto/osteogenic differentiation capacity of SCAPs can be regulated by many factors* in vitro*, including proinflammatory cytokines [1, 4], estrogen [5], and inorganic substance [2, 6].

Diabetes mellitus (DM) is a chronic metabolic disease which is characterized by hyperglycemia and can result in some complications including cardiovascular diseases, nephropathy, osteoporosis, and oral diseases [7]. Diabetic patients have an increased risk of suffering osteopenia and osteoporosis, especially with the uncontrolled hyperglycemia [8, 9]. The development of periodontitis is attributed to the hyperglycemia, in which periodontium and alveolar bone are targets of diabetic damage [10, 11]. Pathologic calcifications such as diffuse calcification and pulp stones always appear in dental pulp tissues of diabetic patients [12]. The underlying mechanisms of bone fracture caused by DM, bone formation, and remodeling in its healing process were still poorly known. Thus, an understanding of the relationship between DM-induced high glucose conditions and the osteogenic differentiation of MSCs is urgently needed [13]. Austin et al. have demonstrated that the function of MSCs has a high dependence on glucose and glucose metabolism [14]. Some studies have shown that high glucose medium is a vital prerequisite for the differentiation of embryonic stem cells (ESCs) into cardiac myocytes [15]. Rat bone marrow mesenchymal stromal cells (rBMMSCs) form increased hard tissues indicated by the osteocalcin production and calcium deposition under a high glucose concentration (over 8.0 mM) [16]. Some studies have revealed that high glucose can reduce proliferation of rat BMMSCs [17] but enhance the adipogenic differentiation of mouse BMMSCs [18], human osteosarcoma cell MG63 [19], and human muscle-derived stem cells [20].

Our previous work has revealed that some signaling pathways participate in the odonto/osteogenic differentiation of SCAPs, including nuclear factor kappa B (NF-*κ*B) pathway and mitogen-activated protein kinase (MAPK) pathway [2, 5]. Moreover, NF-*κ*B is an intracellular transduction signaling pathway found nearly in all cell types, which can be triggered by various activators, i.e., trauma, inflammatory factors, and mineral trioxide aggregate (MTA) [21–23]. It has been proved to take part in the regulation of some biological activities including development, immune response, inflammation, and wound healing [24, 25]. Furthermore, it is extensively involved in the differentiation of osteoblast and osteoclast lineages during the process of tooth eruption and orthodontic tooth movement [22, 26]. To date, there is little information regarding how high glucose exerts an effect on the proliferation and differentiation of SCAPs. In this study, SCAPs were cultured in high glucose media and the effect of high glucose on the odonto/osteogenic differentiation of SCAPs was subsequently investigated. Meanwhile, in the involvement of NF-*κ*B, the potential pathway was also extensively evaluated.

## 2. Materials and Methods

### 2.1. Cell Isolation, Culture, and Identification

This study was approved by Ethical Committee of Stomatological School of Nanjing Medical University (date of approval: 2009-1-1, reference no. 200900128). The young third molars with no caries and periodontic diseases were collected with informed consents from donors (17–20 years old) at the Oral and Maxillofacial Surgery Department of Jiangsu Provincial Stomatological Hospital, following the approved guidelines set by Ethical Committee of Stomatological School of Nanjing Medical University. The apical papilla complexes were gently obtained from the immature dental roots, disposed into 1 mm^3^, and handled with type I collagenase (2 mg/mL) and dispase (4 mg/mL) for 1 h at 37°C. Next, single-cell suspension was cultured into cell culture dishes at the density of 1 × 10^5^ cells/mL and incubated in *α*-MEM supplemented with 10% fetal bovine serum (FBS), 100 U/mL penicillin, and 100 U/mL streptomycin. When reaching 80% confluence, the cells were digested and passaged at the ratio of 1:3. Then, SCAPs were purified according to the standard procedures for magnetic activated cell sorting (MACS) as previously described [27, 28]. Immunocytochemical staining against STRO-1 was used to identify and confirmed the obtained SCAPs and the cells were routinely observed under the phase-contrast inverted microscope. SCAPs at 3-5 passages were used in the following experiments.

### 2.2. MTT (3-(4,5-Dimethylthiazol-2-yl)-2,5-Diphenyl-2,5-Tetrazoliumbromide) Assay

To study the effects of high glucose on the proliferation capacity of SCAPs, the cells at passage 3 were seeded into 96-well plates (Corning, Life Sciences) at a density of 2 × 10^3^ cells/well for 24 h, starved in serum-free media for another 24 h, and, respectively, cultured in normal *α*-MEM (containing 5 mmol/L glucose) and high glucose concentration *α*-MEM (containing 25mmol/L). After 0, 1, 3, 5, and 7 days of culture, 20*μ*l fresh MTT solution (5 mg/ml, Sigma-Aldrich) was added into the wells of each group and incubated for 4 h at 37°C. Culture media were eliminated and formazan was dissolved in 150 *μ*l/well dimethyl sulfoxide (DMSO, Sigma). The absorbance (OD value) was measured at the wavelength of 490 nm using a microtiter plate reader (Titertek, Helsinki, Finland).

### 2.3. Alkaline Phosphatase (ALP) Activity

SCAPs were seeded into the 96-well plates (Corning, Life Sciences) at a density of 2 × 10^3^ cells/well and, respectively, cultured in normal glucose concentration media (containing 5 mmol/L glucose) or high glucose concentration media (containing 25 mmol/L glucose). At days 3 and 7, ALP activity of each group was measured using an ALP kit (Biosino Bio-technology & Science Inc.) and normalized on the basis of equivalent protein concentrations.

### 2.4. Alizarin Red Staining

SCAPs were separately cultured in four different media solutions ( normal culture media containing 5 mmol/L glucose, high glucose concentration media containing 25 mmol/L glucose, mineralized media (MM) containing 5 mmol/L glucose, and mineralized media containing 25 mmol/L glucose) for 14 days. MM is the mineralization-inducing medium containing *α*-MEM, 10% FBS, 100 U/mL penicillin, 100 g/mL streptomycin, 2 mmol/L L-glutamine (Sigma-Aldrich), 50 mg/L ascorbic acid (Sigma-Aldrich), 10 mmol/L *β*-glycerophosphate (Sigma-Aldrich), and 10 nmol/L dexamethasone (Sigma-Aldrich). Alizarin red staining was performed as previously described [29–31]. To quantify the calcified nodules, the alizarin red was eluted from the stained cells with 1 ml/well 10% cetylpyridinium chloride in 10 mM sodium phosphate (pH7.0). The calcium concentration was calculated as previously reported [29] and the final concentrations were normalized to total protein contents.

### 2.5. Real-Time Reverse Transcriptase-Polymerase Chain Reaction (Real-Time RT-PCR)

To investigate the odonto/osteoblast-related gene changes after the inhibition of NF-*κ*B pathway, SCAPs were cultured in normal culture media containing 5 mmol/L glucose or high glucose concentration media containing 25 mmol/L glucose or high glucose concentration media containing 25 mmol/L glucose + BMS345541. The total cellular RNA was extracted by adding TRIzol reagent (Invitrogen, Carlsbad, CA) into cell samples, respectively, according to the manufacturer's instructions. The mRNA was reversed using a PrimeScript RT Master Mix Kit (TaKaRaBiotech., Japan). Real-time RT-PCR was performed by using SYBR® Premix Ex Taq™ kit (TaKaRaBiotech., Japan) and ABI 7300 real-time PCR system. Primers used in this experiment were exhibited in Table 1.* GAPDH* was used as an internal control. The genes expression was calculated by the 2^−△△CT^ method as previously reported [30].

### 2.6. Western Blot Analysis

In order to investigate the effects of high glucose on the odonto/osteogenic differentiation of SCAPs, SCAPs cultured in 5 mmol/L glucose and 25 mmol/L high glucose medium were, respectively, collected at days 3 and 7 and then lysed in RIPA lysis buffer (Beyotime, China). To detect the expression of NF-*κ*B pathway-related proteins, SCAPs treated by 25 mmol/L high glucose for 0 min, 15 min, 30 min, and 60 min were, respectively, harvested to get the cytoplasmic and nuclear proteins with a Keygen Kit (Keygen Bio-tech, Nanjing, China). The same amount of protein was placed into a 10% sodium dodecyl sulfate–polyacrylamide gel electrophoresis (SDS-PAGE) and transferred onto 0.22 *μ*m polyvinylidene fluoride (PVDF, Millipore) membranes. Then, the transferred membranes were blocked in 5% BSA at room temperature for 2 h and incubated with primary antibodies including DSP (sc-33586, Santa Cruz), RUNX2 (ab76956, Abcam, UK), OSX (ab22552, Abcam, UK), OCN (ab93876, Abcam, UK), ALP (ab95462, Abcam, UK), P65(#8242, Cell Signaling Technology), p-P65(#3033, Cell Signaling Technology), I*κ*B*α*(#4814, Cell Signaling Technology), p-I*κ*B*α*(#2859, Cell Signaling Technology), H3(#9728, Cell Signaling Technology), and *β*-ACTIN(AP0060, Bioworld) overnight at 4°C. *β*-ACTIN and H3 served as the internal controls. Finally, the membranes were washed with PBST three times followed by incubation with the secondary antibodies for 1 h at 37°C and then visualized with Image Quant LAS4000 system (GE Healthcare).

### 2.7. Statistical Analysis

Data were calculated by Student's* t*-test.* P* value less than 0.05 was considered to be statistically significant. All the statistical analyses were performed with SPSS 17.0 software (SPSS Inc., Chicago, IL). Image-Pro Plus 5.0 (Media Cybernetics, Inc, Rockville, MD) software was used for the grayscale analysis. All the results were exhibited as means ± SD and experiments were repeated in triplicate.

## 3. Results

### 3.1. Effects of High Glucose on the Proliferation of SCAPs

**Figure 1:**
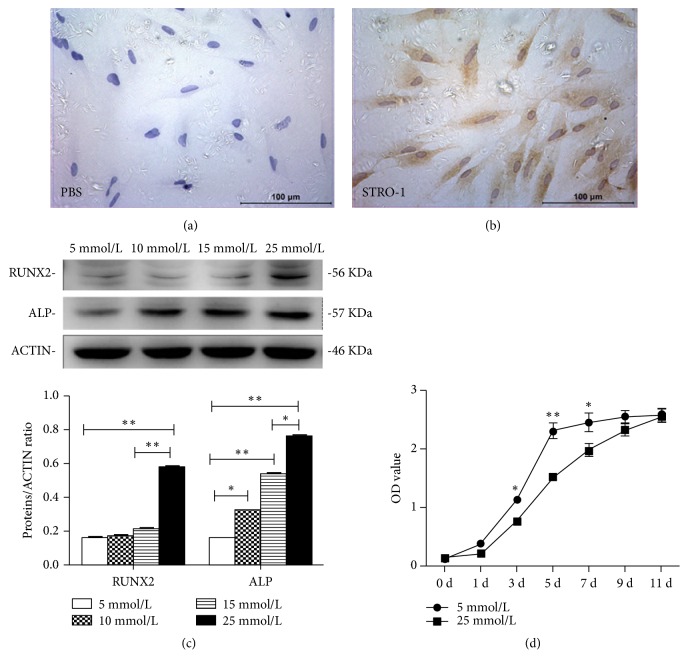
Effects of high glucose on proliferation of SCAPs. (a) Immunocytochemical staining against PBS as negative control. (b) Immunocytochemical staining against STRO-1. (c) Protein expression of RUNX2 and ALP was enhanced with the increased glucose concentrations from 5 mmol/L to 25 mmol/L, in which the highest level was detected in 25 mmol/L group. *∗P *< 0.05, *∗∗ P *< 0.01. (d) MTT assay showed that OD values of SCAPs in 25 mmol/L group were significantly lower than those in control group at days 3, 5, and 7. Values were presented as means ± SD, n = 6, *∗P *< 0.05; *∗∗ P *< 0.01.

SCAPs at passage3 were fibroblast-like (Figure 1(a)) and purified SCAPs were positive against STRO-1 (Figure 1(b)). Western blot assay showed that the expression of osteo/odontogenic markers (RUNX2 and ALP) was gradually upregulated accompanying with the increased glucose concentrations from 5mmol/L to 25mmol/L, and the highest level was in 25mmol/L group. Then 25mmol/L glucose was used in the following experiments (Figure 1(c)). MTT assay showed the OD values of SCAPs cultured in high glucose media were significantly lower than those in normal media at days 3, 5, and 7 (values were presented as means ± SD, n = 6,* P *< 0.05 or* P *< 0.01, Figure 1(d)), indicating that the proliferation ability of SCAPs was inhibited by high glucose.

### 3.2. Effects of High Glucose on the Odonto/Osteogenic Differentiation of SCAPs

**Figure 2:**
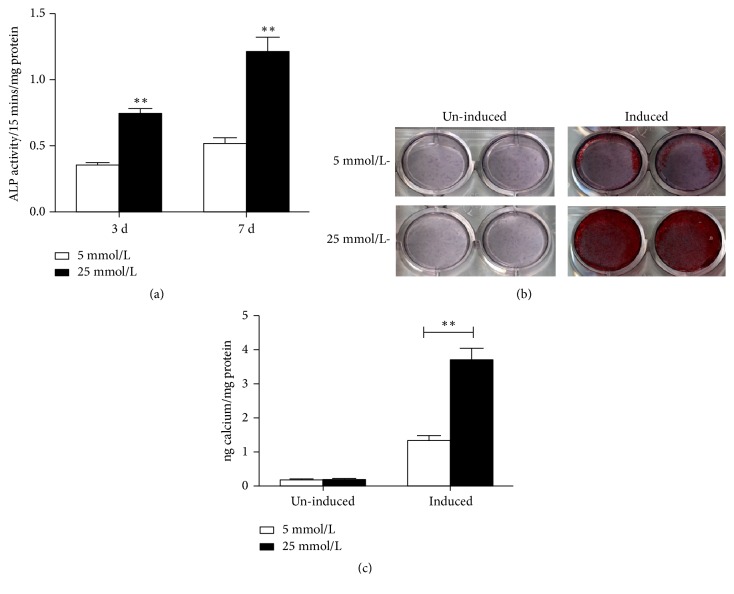
Effects of high glucose on mineralization of SCAPs. (a) ALP activity of SCAPs cultured in 25 mmol/L group was higher than those in 5 mmol/L group at days 3 and 7. Values were presented as means ± SD, n = 6 *∗∗P *< 0.01. (b) Alizarin red staining showed that the calcified nodules formed in uninduced media (without mineralized media) has no significant difference between 25 mmol/L group and control group. However, SCAPs in 25 mmol/L group produced more calcified nodules than those in 5 mmol/L group, when cultured in the mineralize-induced media. (c) Quantitative calcium assay demonstrated the higher calcium deposition in 25 mmol/L group as compared with that in 5 mmol/L group. Data were presented as means± SD, n = 3. *∗∗P *< 0.01.

**Figure 3:**
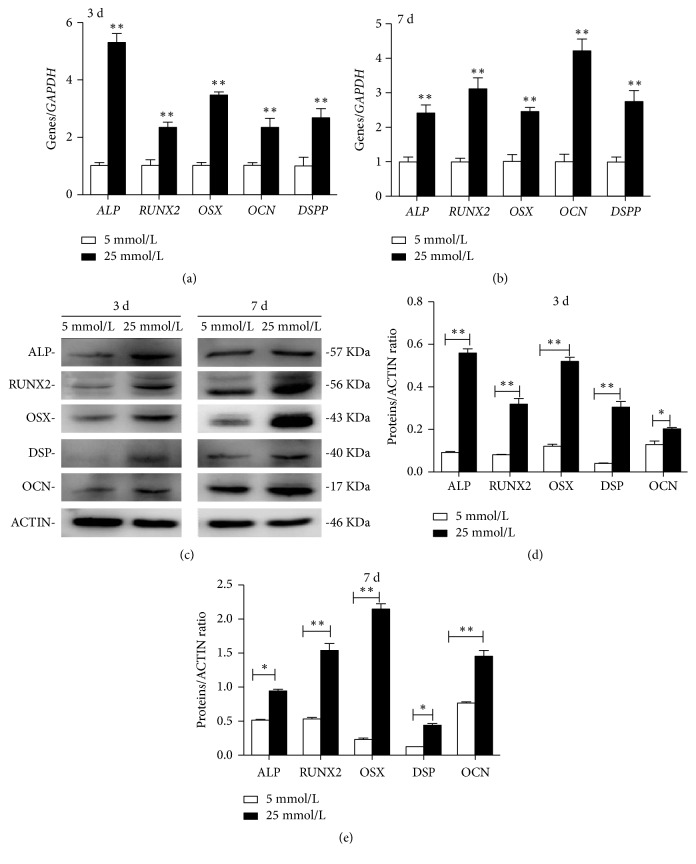
Effects of high glucose on the odonto/osteogenic differentiation of SCAPs. (a, b) The expression of odonto/osteogenic genes (*ALP*,* RUNX*2,* OSX*,* OCN*, and* DSPP*) in 25 mmol/L high glucose-treated SCAPs was significantly higher than that in 5 mmol/L group at days 3 and 7.* GAPDH* served as an internal control. Values were presented as means± SD, n = 6. *∗∗P *< 0.01. (c) Western blot showed the odonto/osteogenic proteins (e.g., ALP, RUNX2, OSX, OCN, and DSP) in 25 mmol/L group were significantly higher than those in 5 mmol/L group at days 3 and 7. ACTIN served as an internal control. (d) Grayscale analysis of panel C. *∗P *< 0.05; *∗∗P *< 0.01.

ALP activity of SCAPs cultured in high glucose was significantly higher than those in 5mmol/L glucose at days 3 and 7 (Figure 2(a),* P *< 0.01). Alizarin red staining assay showed that SCAPs in MM+25 mmol/L glucose group formed more calcified nodules than those in MM+5mmol/L glucose group (Figure 2(b)). Meanwhile, CPC quantitative calcium assay demonstrated the higher calcium deposition in MM+25mmol/L glucose group than MM+5 mmol/L glucose group (Figure 2(c),* P *< 0.01), indicating that high glucose can enhance the mineralization capacity of SCAPs. Real-time RT-PCR showed that the expression of* ALP*,* RUNX*2,* OSX*,* OCN*, and* DSPP* was enhanced in high glucose-treated SCAPs at day 3 (Figure 3(a)) or day 7 (Figure 3(b)) as compared with that in control group. Western blot results demonstrated that the expression of odonto/osteogenic proteins (ALP, RUNX2, DSP, OSX, and OCN) was significantly increased after the treatment of 25mmol/L glucose for 3 and 7 days as compared with that in control group (Figures 3(c), 3(d), and 3(e)).

### 3.3. NF-*κ*B Pathway Involvement in High Glucose-Mediated Odonto/Osteogenic Differentiation of SCAPs

**Figure 4:**
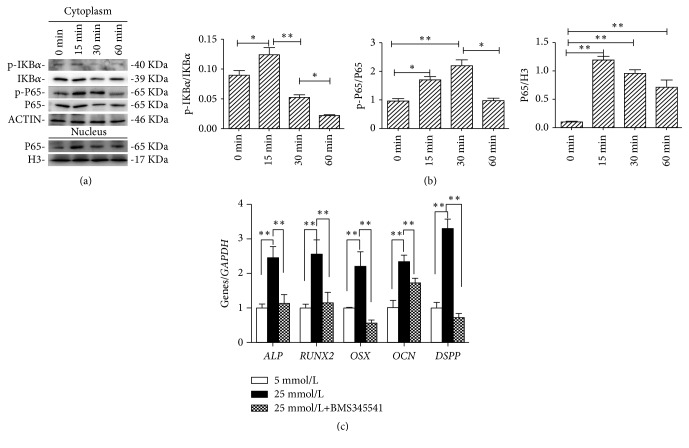
NF-*κ*B pathway involvement in high glucose-mediated odonto/osteogenic differentiation of SCAPs. (a) The protein levels of P65, p-P65, I*κ*B*α*, and p-I*κ*B*α* in the cytoplasm of 25 mmol/L high glucose-treated SCAPs at 0, 15, 30, and 60 min, respectively. *β*-ACTIN served as an internal control. The nuclear P65 expression of 25 mmol/L high glucose-treated SCAPs at 0, 15, 30, and 60 min, respectively. Histone 3 served as an internal control. (b) Quantitative analysis for the ratios of p-I*κ*B*α*/I*κ*B*α* and p-P65/P65 and grayscale analysis of panel A after 25 mmol/L glucose treatment at indicated time points. Values were presented as means± SD, n = 6. *∗ P*< 0.05; *∗∗P*< 0.01. (c) The expression of odonto/osteogenic genes (*ALP*,* RUNX*2,* OSX*,* OCN*, and* DSPP*) in 25 mmol/L+BMS345541 group was obviously decreased as compared with that in 25 mmol/L group. Values were presented as means ± SD, *∗∗*2^−∆∆Ct^ ≥ 2,* P*< 0.01; *∗* 1 < 2^−∆∆Ct^ < 2,* P *< 0.05, n=3.

NF-*κ*B pathway proteins changed after the treatment of high glucose for 15, 30, and 60 min. The expression of p-I*κ*B*α* increased at 15min and then decreased. Cytoplasmic p-P65 protein expression was obviously upregulated at 15 min and slowly decreased, while the protein expression ofnulearP65 was increased at 15 min and then gradually decreased at 30 min and 60 min (*P*< 0.01, Figures 4(a) and 4(b)). To confirm the role of NF-*κ*B pathway in high glucose-mediated odonto/osteogenic differentiation of SCAPs, BMS345541 (IKK inhibitor) was used to inhibit NF-*κ*B pathway prior to the high glucose treatment. Real-time RT-PCR results showed that the expression of odonto/osteogenic genes including* ALP*,* RUNX*2,* OSX*,* OCN*, and* DSPP *was significantly downregulated, as compared with that in high glucose-treated SCAPs (*P*< 0.01, Figure 4(c)).

## 4. Discussion

Patients with DM may suffer some skeletal disorders including osteoporosis, periodontal diseases, and the diabetic foot syndrome, indicating that DM is associated with specific alterations of bone metabolism [32]. In the context of diabetogenesis, the key biological issues are how bone tissues originate, proliferate, and differentiate [8]. Recruitment of an adequate amount of MSCs to the microenvironment around the bone injury is essential for effective bone repair [33]. It is well known that SCAPs are the most important stem cells for dental tissue regeneration [4]. Thus, it will make sense to investigate the proliferation and odonto/osteogenic differentiation of SCAPs under a high glucose microenvironment.

In the present study, STRO-1^+^ SCAPs were successfully isolated and MTT assay showed that 25mmol/L high glucose reduced the proliferation ability of SCAPs. Mechanistically, high glucose environment may induce an adaptive response to the increased oxidative stress and promote reactive oxygen species (ROS) removal, resulting in a decreased proliferation of stem cells [34, 35]. Some studies have demonstrated that high glucose can inhibit the proliferation ability of BMSCs by activating GSK3*β* to suppress cyclin D1 and CXCR-4 [36].

High glucose has the ability to attenuate the differentiation capacity of ESCs into neurocytes [37], BMSCs, and hPDLSCs into osteoblasts [34, 36]. In contrast, high glucose can promote the osteogenic differentiation capacity of human periodontal ligament fibroblasts [38]. To date, effects of high glucose on the odonto/osteogenic differentiation of SCAPs are still not determined. This study demonstrated for the first time that 25 mmol/L high glucose can enhance the odonto/osteogenic differentiation of SCAPs. Previous studies have found the appearance of pulp stones and thickened predentin in diabetic rat [39, 40]. Moreover, positive correlation between Type II diabetes mellitus and pulp stones has been revealed through analyses of various clinical cases [41]. It has been reported that advanced glycation end-products (AGE) can stimulate the osteogenic differentiation, which is verified by the upregulation of ALP activity, osteopontin (OPN), and osteocalcin (OCN) in rat dental pulp cells [39].

An increase in osteogenic differentiation level after the high glucose treatment was indicated by an upregulation of several odonto/osteogenic markers including* ALP*/ALP,* RUNX*2/RUNX2,* DSPP*/DSP,* OSX*/OSX, and* OCN*/OCN[42].* ALP*/ALP is mainly generated by osteoblasts and was thought to participate in the degradation of pyrophosphate to provide sufficient inorganic phosphate for the occurrence of mineralization [43].* RUNX*2/RUNX2 and* OSX*/OSX are both important transcription factors necessary for the osteogenic differentiation [44], in which* OSX* is a downstream gene of* RUNX*2 and regulated by* RUNX*2 [45].* OCN*/OCN is highly presented in the bone extracellular matrix and actively involved in bone formation [46]. As the specific marker of the odontogenesis,* DSPP*/DSP mainly appears in the dentin or predentin structures [47, 48]. Alkaline phosphatase (ALP) activity and alizarin red staining confirmed the enhanced odonto/osteogenic differentiation ability of SCAPs in high glucose media.

In this study, NF-*κ*B signaling pathway was activated after high glucose treatment and the increased odonto/osteogenic capability of SCAPs can be blocked by the NF-*κ*B signaling pathway inhibitor (BMS345541). Various studies have proved that high glucose can activate NF-*κ*B signaling in different cells [21, 23, 49–51]. Some reports have revealed that reactive oxygen species (ROS) is a crucial signal driving the differentiation of both adipose-derived stem cells (ADSCs) and myeloid-derived suppressor cells (MDSCs) into adipocytes after high glucose exposure. ROS may modulate the activity of transcription factors directly, such as NF-*κ*B, thus changing the related gene expression [20, 52]. Moreover, high glucose can induce the expression as well as the secretion of Par-4 in islet beta cells while TLR-2/4 expression is significantly upregulated in retinal microvascular endothelial cells after high glucose treatment, which subsequently causes the activation of NF-*κ*B [53–55].

**Figure 5:**
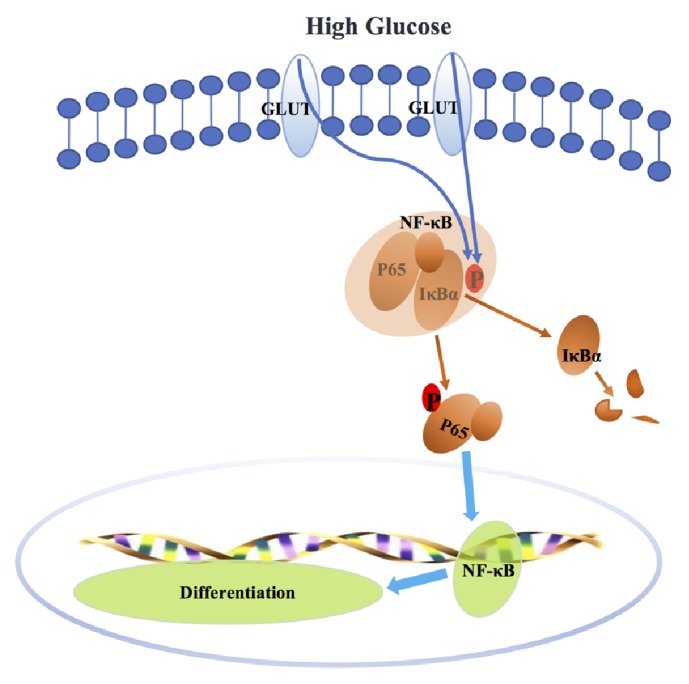
Schematic diagram for the activation of NF-*κ*B pathway by high glucose. High glucose is absorbed by SCAPs mainly by glucose transporters (GLUT). High glucose can induce the phosphorylation of IkB*α* in the cytoplasm, which subsequently leads to the rapid hydrolysis of IkB*α*. P65 is then phosphorylated and transported into nuclei, which finally causes the activation of NF-*κ*B pathway and odonto/osteogenic differentiation of stem cells.

Together, high glucose is transported into SCAPs by glucose transporters (GLUT) [56], and then I*κ*B*α* is degraded and released from the p50/p65 complex, which leads to the translocation of P65 into nuclei and the activation of NF-*κ*B. The odonto/osteogenic genes are expressed later which ultimately result in the committed differentiation of SCAPs (Figure 5).

## 5. Conclusions

In summary, high glucose could reduce the proliferation ability of SCAPs. Meanwhile, the odonto/osteogenic differentiation capacity of SCAPs was enhanced in high glucose environment by activation of NF-*κ*B pathway. This work provides a novel insight into the interactions between high glucose and apexogenesis mediated by SCAPs, in which high glucose may have a crucial influence on the odontogenesis and pulp calcification [40, 41, 56]. More extensive investigations should be performed to explore the potential applications of high glucose in clinical endodontic treatments.

## Figures and Tables

**Table 1:** Sense and antisense primers for real-time reverse transcription polymerase chain reaction.

Genes	Primers	Sequences (5'-3')
*ALP*	Forward	GACCTCCTCGGAAGACACTC
	Reverse	TGAAGGGCTTCTTGTCTGTG
*RUNX*2	Forward	TCTTAGAACAAATTCTGCCCTTT
	Reverse	TGCTTTGGTCTTGAAATCACA
*DSPP*	Forward	ATATTGAGGGCTGGAATGGGGA
	Reverse	TTTGTGGCTCCAGCATTGTCA
*OSX*	Forward	CCTCCTCAGCTCACCTTCTC
	Reverse	GTTGGGAGCCCAAATAGAAA
*OCN*	Forward	AGCAAAGGTGCAGCCTTTGT
	Reverse	GCGCCTGGGTCTCTTCACT
*GAPDH*	Forward	GAAGGTGAAGGTCGGAGTC
	Reverse	GAGATGGTGATGGGATTTC

## Data Availability

The data used to support the findings of this study are available from the corresponding author upon request.
